# Supplementary material for: Lupinus albus γ-Conglutin: New Findings about Its Action at the Intestinal Barrier and a Critical Analysis of the State of the Art on Its Postprandial Glycaemic Regulating Activity
Source: Nutrients. 2022 Sep 5;14(17):3666. doi: 10.3390/nu14173666 (PMC9460565; doi:10.3390/nu14173666)
Supplement: Supplementary file 1 [file nutrients-14-03666-s001.zip › nutrients-1878397-supplementary.pdf]

nutrients

MDPI

*Supplementary materials*

# *Lupinus albus* γ-conglutin: new findings about its action at the intestinal barrier and a critical analysis of the state of the art on its postprandial glycaemic regulating activity

**Giuditta C. Heinzl [1,*], Marco Tretola [2] , Stefano De Benedetti [1], Paolo Silacci [3] and Alessio Scarafoni [1]**

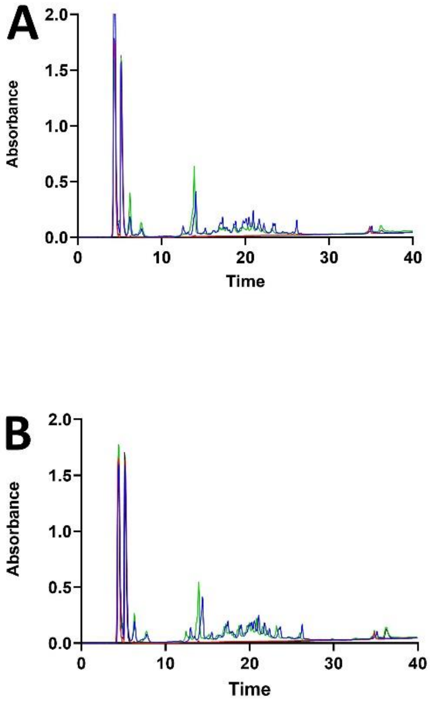


**Supplementary Figure S1**. RP-HPLC chromatograms of jejunum (A) and ileum (B) apical and basolateral sides of Ussing chamber at the beginning of the experiment (blue and red lines, respectively) and after 30 min of incubation with γ-C50 sample (green and black lines, respectively). Intestinal enzymatic activity on γ-C sample.

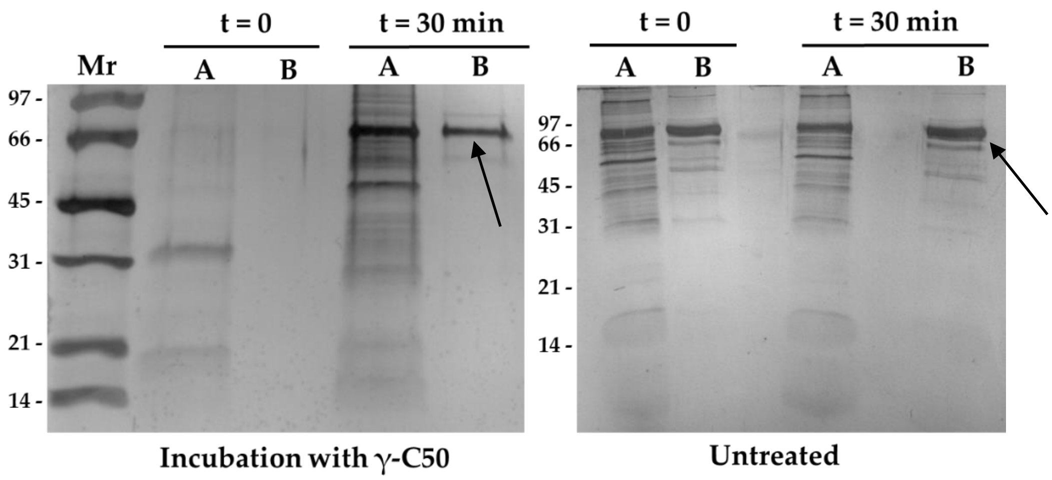


```
1   MSIHQPNPTS RVKHRRKLQW THPQVAQLLK KKKKKRMLGI VNIQRCNTRW HSGPSSAYSR GVFRRDTYKS EIAHRFKDLG 80
81  EQYFKGLVLI AFSQHLQQCP YEEHVKLVRE VTEFAKTCVA DESAENCDKS IHTLFGDKLC AIPSLREHYG DLADCCEKEE 160
161 PERNECFLQH KNDNPDIPKL KPDPVALCAD FQEDEQKFWG KYLYEIARRH PYFYAPELLY YAIIYKDVFS ECCQAADKAA 240
241 CLLPKIEHLR EKVLTSAAKQ RLKCASIQKF GERAFKAWSL ARLSQRFPKA DFTEISKIVT DLAKVHKECC HGDLLECADD 320
321 RADLAKYICE NQDTISTKLK ECCDKPLLEK SHCIAEAKRD ELPADLNPLE HDFVEDKEVC KNYKEAKDVF LGTFLYEYSR 400
401 RHPDYSVSLL LRIAKIYEAT LEDCCAKEDP PACYATVFDK FQPLVDEPKN LIKQNCELFE KLGEYGFQNA LIVRYTKKVP 480
481 QVSTPTLVEV ARKLGLVGSR CCKRPEEERL SCAEDYLSLV LNRLCVLHEK TPVSEKVTKC CTESLVNRRP CFSALTPDET 560
561 YKPKEFVEGT FTFHADLCTL PEDEKQIKKQ TALVELLKHK PHATEEQLRT VLGNFAAFVQ KCCAAPDHEA CFAVEVLDLF 640
641 VFILMGPVSF VVAFDQART                                                                   659
```
The matched peptides cover **6%** (44/659 AA's) of the protein.

Protein Name: **Albumin**
Species: **PIG**
**SwissProtSus14dec21.PIG** Accession #: **A0A287BAY9**
MS Digest Index #: **12766**
Masses are:
pI of Protein: **7.21**
Protein MW: **75774.0** Da

**Supplementary Figure S2**. SDS-PAGE of the polypeptides composition present in the apical (A) and basolateral (B) side of Ussing chamber following incubation with γ-C50 sample for 30 min. The 65 kDa band present in the samples has been excised and sent to mass spectroscopy. Below the MS identification of the polypeptide indicated with the arrow.

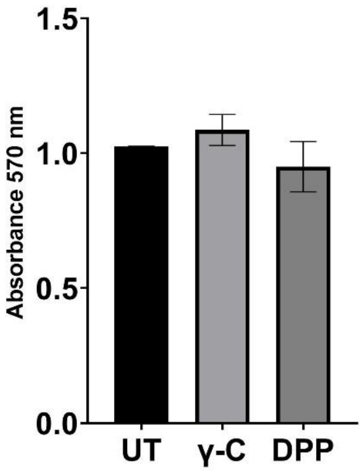


**Supplementary Figure S3**. Vitality of Caco-2 cells after 4 h of treatment with intact γ-Conglutin (γ-C) and digested with pepsin and pancreatin (DPP). Determined with MTT method.

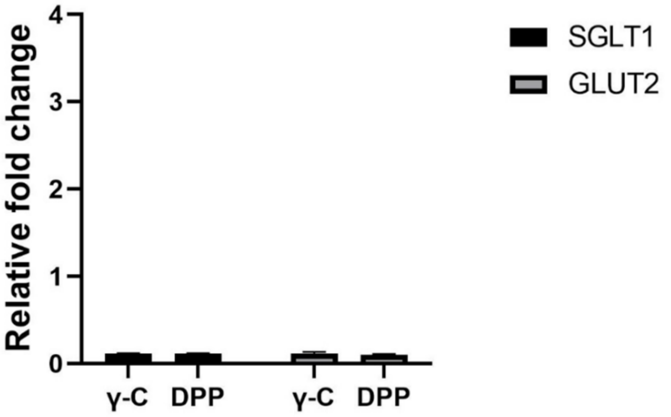


**Supplementary Figure S4**. Effects of the 4 h incubation of differentiated Caco-2 cells with 1 mg/mL (γ-C) and pepsin and pancreatin digested sample (DPP) on SGLT1 and GLUT2 gene expression. Data are expressed as mean ± standard deviation. Non statistically significative differences are evident between the samples.

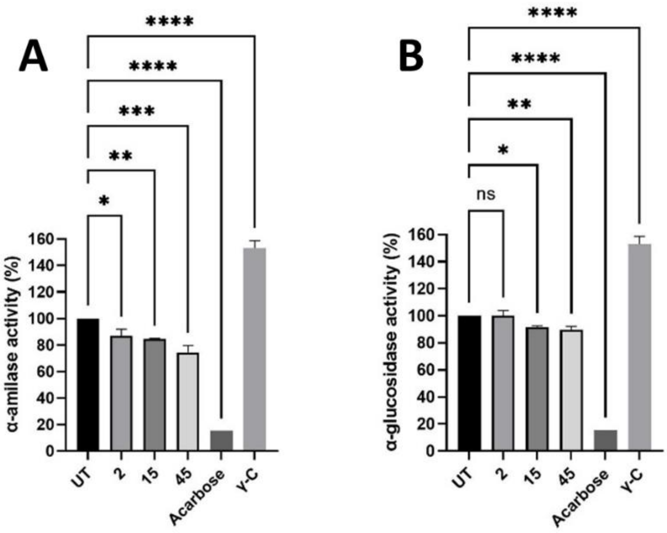


**Supplementary Figure S5**. Effect of γ-C-derived peptides at 2, 15 and 45 min of digestion on $\alpha$-amilase (A) and $\alpha$-glucosidase (B) enzymes activity. Values are mean ± standard deviation of three independent experiments; ****p ≤ 0.0001, ***p ≤ 0.001, **p ≤ 0.01.
